# Genomic and pathogenicity analysis of two novel highly pathogenic recombinant NADC30-like PRRSV strains in China, in 2023

**DOI:** 10.1128/spectrum.00368-24

**Published:** 2024-08-20

**Authors:** Hao Chang, Xiaopeng Gao, Yu Wu, Fang Wang, Minting Lai, Jiaying Zheng, Yingwu Qiu, Yiping He, Xiangjie Liang, Kun Yuan, Limiao Lin, Haishen Zhao, Guihong Zhang, Qunhui Li, Yankuo Sun

**Affiliations:** 1 Key Laboratory of Zoonosis Prevention and Control of Guangdong Province, College of Veterinary Medicine, South China Agricultural University, Guangzhou, China; 2 Wen’s Group Academy, Wen’s Foodstuffs Group Co., Ltd., Xinxing, Guangdong, China; 3 Maoming Branch, Guangdong Laboratory for Lingnan Modern Agriculture, Maoming, China; 4 National Engineering Research Center for Breeding Swine Industry, South China Agricultural University, Guangzhou, China; 5 Guangzhou Yue Xiu animal husbandry food technology limited, Guangzhou, China; University of Prince Edward Island, Charlottestown, Prince Edward Island, Canada

**Keywords:** PRRSV, phylogenetic analysis, recombination analysis, pathogenicity

## Abstract

Porcine reproductive and respiratory syndrome viruses (PRRSVs) exhibit high mutability and recombination, posing challenges to their immunization and control. This study isolated two new PRRSV strains, GD-7 and GX-3, from samples collected in Guangdong and Guangxi in 2023. Whole-genome sequencing, along with phylogenetic and recombination analyses, confirmed that GD-7 and GX-3 are natural novel recombinant strains of NADC30 PRRSV. Moreover, we established a pathogenicity model for piglets and sows based on the two isolates. The results of piglet pathogenicity revealed that both GD-7 and GX-3 caused clinical symptoms such as fever, loss of appetite, depression, and slow weight gain. Moreover, we observed that the mortality rate of GD-7-inoculated group piglets was 33.3%, which was similar to that of piglets infected with other highly pathogenic PRRSV strains and exceeded the mortality rate of most NADC30-like PRRSV. In pregnant sow models, the survival rate of sows in the GD-7 group was 75%, in contrast to the GX-3 group, where no sow mortality was observed, and both strains resulted in abortion, mummified fetuses, and stillbirths. These results highlight the elevated pathogenicity of these recombinant strains in sows, with GD-7 mainly causing sows to abort, and GX-3 mainly causing sows to give birth to mummified fetuses. This study introduces two distinct clinical recombinant PRRSV strains that differ from the prevalent strains in China. This research furthers our understanding of the epidemiology of PRRSV and underscores the significance of ongoing monitoring and research in the face of evolving virus strains. Moreover, these discoveries act as early warnings, underscoring the necessity for active control and immunization against PRRSV.

**IMPORTANCE:**

Since the discovery of NADC30-like PRRSV in China in 2013, it has gradually become the dominant strain of PRRSV in China. NADC30-like PRRSV exhibits high recombination characteristics, constantly recombining with different strains, leading to the emergence of numerous novel strains. Of particular importance is the observation that NADC30-like PRRSV with different recombination patterns exhibits varying pathogenicity, which has a significant impact on the pig farming industry. This emphasizes the necessity of monitoring and responding to evolving PRRSV strains to develop effective immunization and control strategies. In this paper, we conducted pathogenicity studies on the isolated NADC30-like PRRSV and analyzed the differences in the genomes and pathogenicity of the different strains by recording clinical symptoms, temperature changes, detoxification tests, and changes in viremia and histopathology in infected pigs. This was done to provide a theoretical basis for the epidemiological situation and epidemic prevention and control of PRRSV.

## INTRODUCTION

Porcine reproductive and respiratory syndrome (PRRS), commonly known as “blue ear disease,” remains an economically important swine disease caused by the PRRS virus (PRRSV). The disease has been endemic worldwide for more than 35 years since it was first reported as a “mystery swine disease” in 1987 (1, 2). PRRSV, a positive single-stranded, nonsegmented RNA virus, contains spherical viral particles with diameters of 50–60 nm and a capsular structure. It has been classified by the International Committee on Taxonomy of Viruses into the order Nido, family *Arteritis*, and genus *Arteritis* (3). PRRSV exists as two genotypes: genotype I (Eurpobartevirus Betaarterivirus suid 1) exemplified by Lelystad virus and genotype II (Ampobartevirus Betaarterivirus suid 2) represented by VR2332. These genotypes exhibit approximately 60% nucleotide homology (4).

In China, three significant events of PRRSV epidemic strain variations have occurred: first, since the first report of PRRSV in China in 1996 (5), a predominant strain has been the classical CH-1a. Second, in 2006, the emergence of highly pathogenic (HP)-PRRSV (6) resulted in substantial losses to the Chinese pig farming industry. The third event took place in 2013, with the entry of NADC30 PRRSV into China and its recombination with locally prevalent strains, giving rise to NADC30-like PRRSVs characterized by a discontinuous deletion in the NSP2 region (119 + 1 + 19 at amino acids 322–432, 483, and 504–522). Currently, NADC30-like PRRSV prevails in China, with recombination serving as the primary driver of both pathogenicity and genetic diversity. The pathogenicity tends to be intermediate between parental strains, resulting in diverse clinical outcomes ranging from inconsequential to severe symptoms (7). Various NADC30-like PRRSVs can manifest distinct clinical symptoms, presenting a formidable challenge for prevention and control in swine farms (8). Moreover, there remains a paucity of studies on the pathogenicity of NADC30-like strains in both piglets and pregnant sows.

In this study, we conducted genomic analysis on two isolated NADC30-like PRRSVs, GD-7 and GX-3, and performed pathogenicity experiments on piglets and gestating sows. Our results comprehensively demonstrate the pathogenic manifestations of these two emerging strains in piglets and pregnant sows, which has been limited in previous research. This enhances our systematic understanding of the two novel NADC30-like PRRSV strains. Moreover, the findings of this study aim to serve as a reference for epidemiological investigations and the control of PRRSV.

## MATERIALS AND METHODS

### Sample collection and detection

In April 2023, a suspected PRRSV outbreak occurred in a pig farm in Guangdong as well as a pig farm in Guangxi, with clinical manifestations of abortions, stillbirths, and fattening pigs exhibiting significant respiratory diseases. We collected lung tissue from diseased pigs in two farms after obtaining permission from the animal owners. Tissue samples were ground using a freeze grinder (Shanghai Jingxin) and total RNA was extracted from the supernatant using the RNeasy kit (Magen, Shanghai, China) and detected using the real-time polymerase chain reaction (RT-PCR) 2 × AceQ Universal U kit + Probe Master V2 PCR detection kit (Vazyme Biotech, Nanjing, China) and GP5 primers (Table 1).

**TABLE 1:** The GP5 primer sequence used in the RT-PCR in this study

Primer	Amplified fragment	Primer sequences
Detection primers	GP5-F	ACCTGAGACCATGAGGTGGGC
	GP5-R	GCCAGAATGTACTTGCGGCCTA

### Virus isolation and identification

Positive tissue was ground, centrifuged, filtered using a 0.22-µm filter, and inoculated onto primary alveolar macrophages (PAMs) for virus isolation. Cells were cultured in RPMI 1640 media (Fisher Scientific, Waltham, MA, the USA) containing 10% fetal bovine serum at 37°C in a 5% humidified CO_2_ atmosphere. When approximately 80% of virus-infected cells exhibited cytopathic effects (CPE), the virus was harvested by freeze-thawing. The supernatant was collected and inoculated into Marc-145; after successive passaging cultures, isolated strains were identified by immunofluorescence assay (IFA) and agarose gel electrophoresis. IFA experiments were performed using a PRRSV N protein mouse monoclonal antibody prepared in our laboratory and an Alexa Fluor 488 goat anti-mouse immunoglobulin G (Abcam, UK).

### Genome sequencing and analysis

The whole genome of PRRSV GD-7 and GX-3 was amplified using specific primers; moreover, each PCR product was purified using EasyPure Quick Gel Extraction Kit (Transgen, Beijing, China) and sequenced using Songon (Shanghai, China). Untranslated regions were sequenced using 3′-RACE kit and 5′-RACE kit (Songon, Shanghai, China). All the primers that were used to amplify PRRSV translated regions sequence were designed and preserved in our laboratory.

Multiple sequence alignments were analyzed using the DNASTAR software to determine sequence homology. The phylogenetic trees of GX-3 and GD-7 strains were constructed by MEGA 11 with the neighbor-joining method from 1,000 bootstrap replicates for alignment, using multiple sequences of representative PRRSV available in GenBank. Recombinant events were evaluated based on the Recombination Detection Program 4 (RDP4), Chimaera, BootScan, 3Seq, GENECONV, MaxChi, and SiScanto (9). Furthermore, a potential recombination event was considered to have occurred when at least six of the seven detection methods were positive. Similarity comparisons were further performed using Simplot version 3.5.1 within a 500 bp sliding window along the genomic alignment (20 bp step) (10).

### Animal trials for pathogenicity analyses

To assess the pathogenicity of GX-3 and GD-7 PRRSVs, we purchased 18 piglets and 12 sows. Their breed, number, and grouping in the experiment adhered to the 3R principle. All animal experiments performed in this study were approved by the Animal Ethics Committee of South China Agricultural University and adhered to the guidelines of the South China Agricultural University Institutional Animal Care and Use Committee (SCAU-AEC-2023C043). The experiments were conducted in an appropriate laboratory setting that complies with the national requirements and animal welfare principles.

PRRSV antigen- and antibody-negative piglets (*n* = 18; 35 days old) were randomly divided into three groups: GD-7-inoculated piglets [*n* = 6; each piglet intramuscularly injected with 2 mL of GD-7 virus containing a 50% tissue culture infectious dose (TCID_50_) of 2 × 10^5^], GX-3-inoculated piglets (*n* = 6; each piglet intramuscularly injected with 2 mL of GX-3 virus containing 2 × 10^5^ TCID_50_), and a control group of mock-inoculated piglets (*n* = 6; each piglet intramuscularly injected with 2 mL of Dulbecco’s modified Eagle’s media). PRRSV antigen- and antibody-negative sows (*n* = 12; 85 days of gestation) were randomly divided into three groups: GD-7-inoculated sows (*n* = 4; each sow intramuscularly injected with 2 mL of GD-7 virus containing 2 × 10^5^ TCID_50_), GX-3–inoculated sows (*n* = 4; each sow intramuscularly injected with 2 mL of GX-3 virus containing 2 × 10^5^ TCID_50_), and a control group of mock-inoculated sows (*n* = 4; each sow intramuscularly injected with 2 mL of Dulbecco’s modified Eagle’s media). Each group was independently reared with *ad libitum* access to feed and water.

Following inoculation, daily monitoring of pig body temperature, body weight, and feed intake was conducted, and clinical signs were also recorded. Serum was collected from piglets on days 0, 3, 7, and 14 post-inoculation and from sows on days 1, 3, 7, 11, 14, 21, 28, 40, and 49. PRRSV N-specific antibodies were tested using the commercial IDEXX HerdChek PRRS X3 Enzyme-linked Immunosorbent Assay Kit (Westbrook, ME, USA). Viral copy number was determined through reverse-transcription polymerase chain reaction (RT-PCR) of the collected serum samples to monitor detoxification and detect viremia. Furthermore, sow farrowing data, including total litter size, healthy litter size, and abortions, were recorded. Piglets were euthanized 14 days after disease onset and then dissected. Subsequently, lung samples and submandibular lymph nodes were collected, fixed using 4% paraformaldehyde, subjected to paraffin-embedded sectioning, and stained with hematoxylin and eosin (H&E) for histopathology observation under a light microscope. Partial samples of the heart, liver, spleen, lungs, kidneys, submandibular lymph nodes, thymic lymph nodes, and inguinal lymph nodes of the piglets were also collected following autopsy and tested for toxin load *via* RT-PCR.

### Statistical analysis

Experimental data were analyzed for significance using GraphPad Prism version 5.0 and were presented as mean ± standard deviation. *P*-values of <0.05 were considered to indicate statistical significance, and *P*-values of <0.01 and <0.001 were considered highly significant.

## RESULTS

### Virus isolation and identification

**Fig 1:**
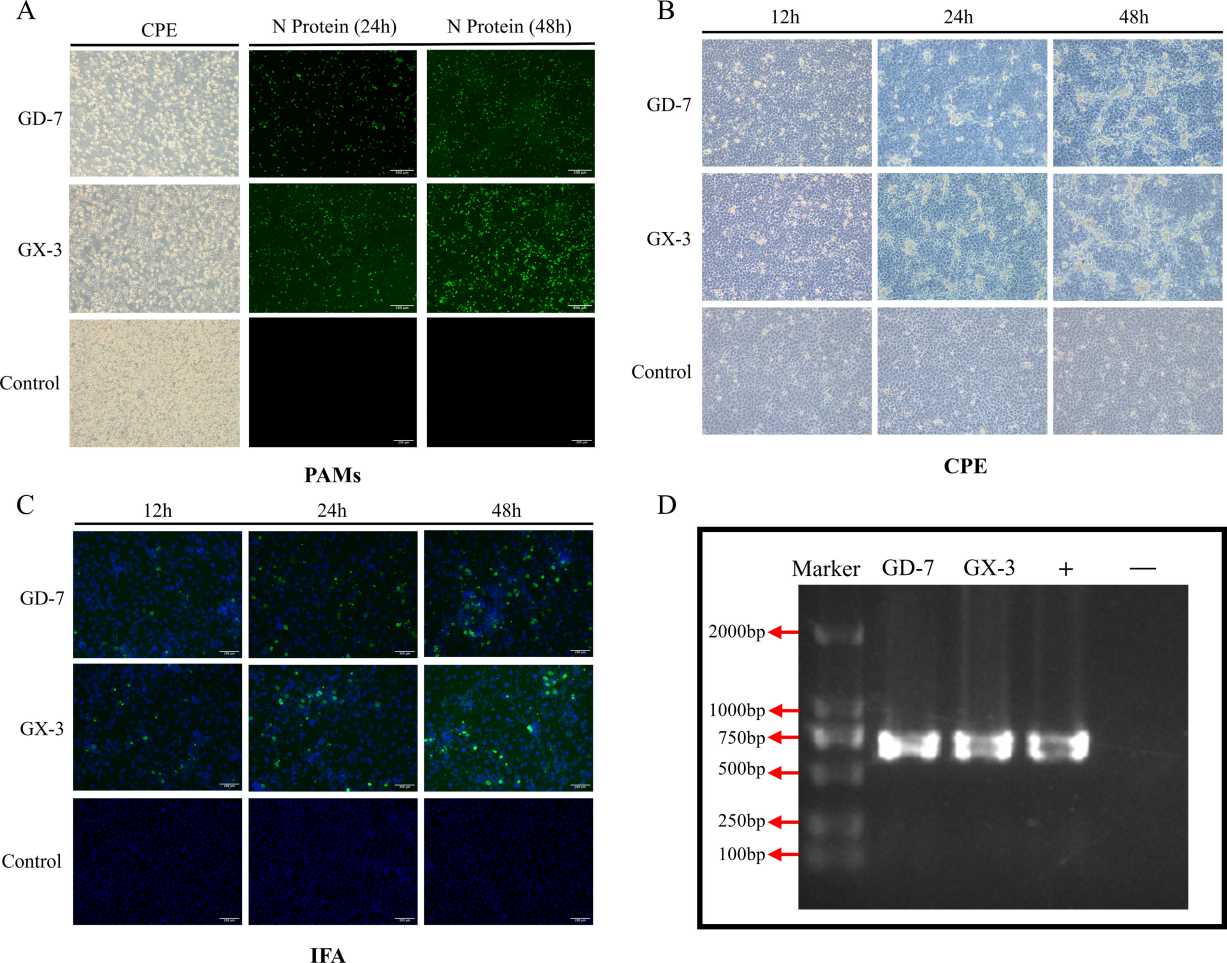
Isolation and characterization of viruses. (**A**): Lesions in PAM cells after inoculation with GX-3 and GD-7 disease material and detection of PRRSVN protein by IFA. (**B**): Cytopathic conditions of MARC-145 at 24, 36, and 72 h post-infection (hpi). (**C**): FAs show the reactivity of a monoclonal antibody against PRRSV N protein to GD-7 and GX-3 strains infected at 24, 36, and 72 hpi. (**D**): Lesion MARC-145 cell nucleic acid was collected and detected by agarose gel electrophoresis after PCR amplification, + is the positive control, − is the blank control.

**Fig 2:**
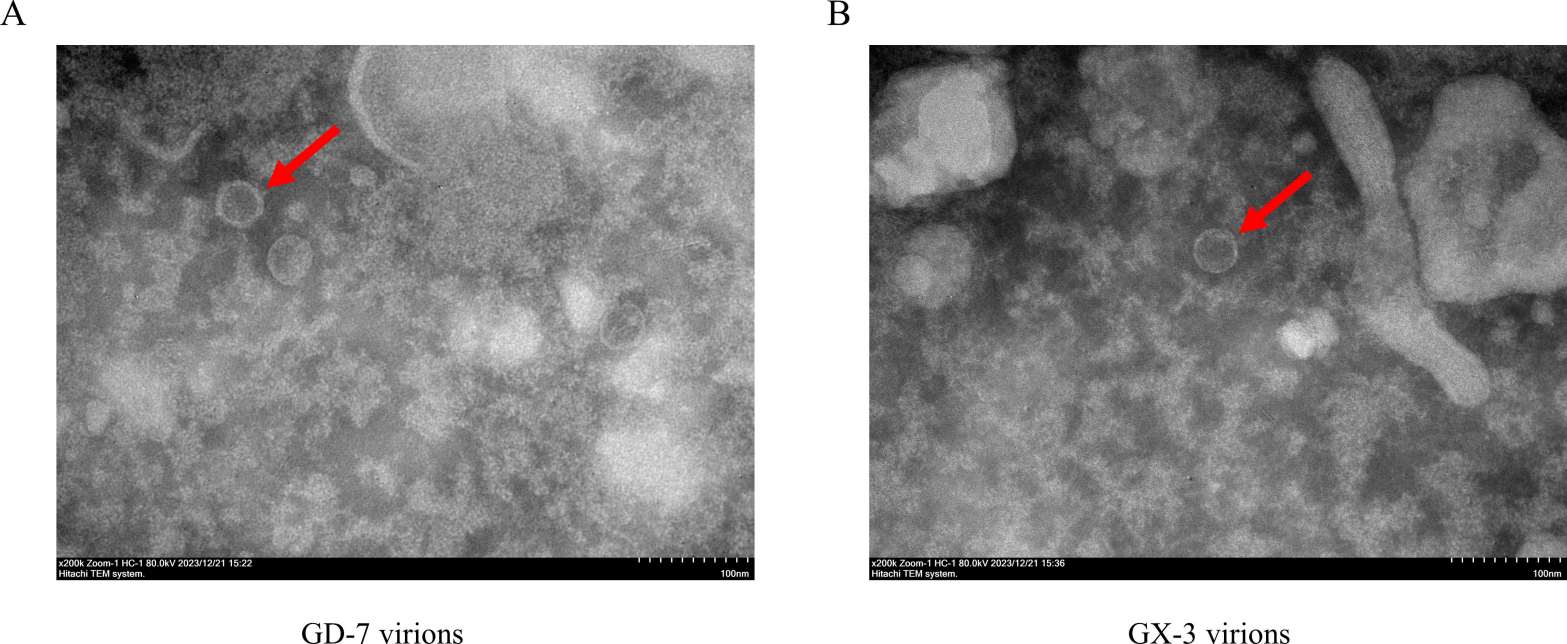
Observation of viral particles. (**A**) Virus particles of the GD-7 strain were observed by electron microscopy. Magnification ×200K. (**B**): Virus particles of the GX-3 strain observed by electron microscopy. Magnification ×200K.

The presence of PRRSV was initially confirmed by inoculating the diseased material onto porcine alveolar macrophage cells, observing cell lesions, and conducting IFA (Fig. 1A). Subsequently, viral fluids were collected, inoculated into MARC-145 cells, and subjected to continuous culture, resulting in the acquisition of two PRRSV strains. The biological characteristics of the strains were evaluated, which revealed that cell lesions, including cell death and detachment, became more apparent with prolonged infection time (Fig. 1B). Meanwhile, IFA showed an enhanced fluorescence-positive signal over time (Fig. 1C). Nucleic acids from lesional MARC-145 cells were collected, PCR-amplified, and examined *via* agarose gel electrophoresis, confirming the expected size of the target fragment (Fig. 1D). In addition, virus particles were observed using an electron microscope after ultracentrifugation (Fig. 2). In summary, two PRRSV strains GD-7 and GX-3 were obtained through isolation and characterization.

### Genome sequencing and analysis

**Fig 3:**
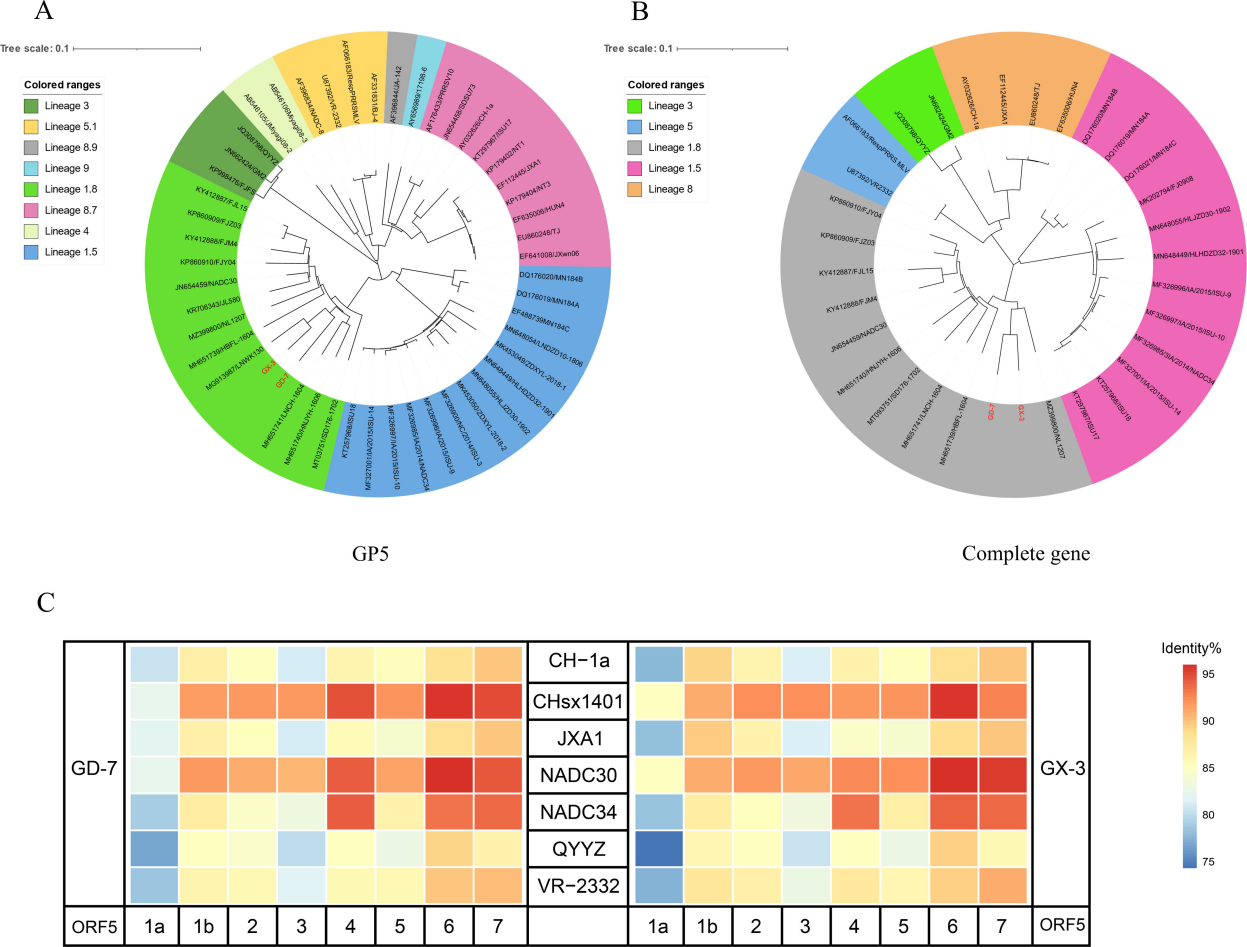
Genome analysis and recombination analysis. (**A**) The phylogenetic tree was constructed based on the GP5 gene of GX-3 and GD-7 strains and 47 reference PRRSV strains. (**B**) Phylogenetic trees were constructed based on the complete genome of the GX-3 and GD-7 strains with 30 representative reference PRRSV strains. Phylogenetic trees were constructed using the distance-based neighbor-joining method with 1,000 bootstrap replicates in MEGA11. (**C**) Heat map constructed based on the homology of the isolated strain with other reference strains.

**Fig 4:**
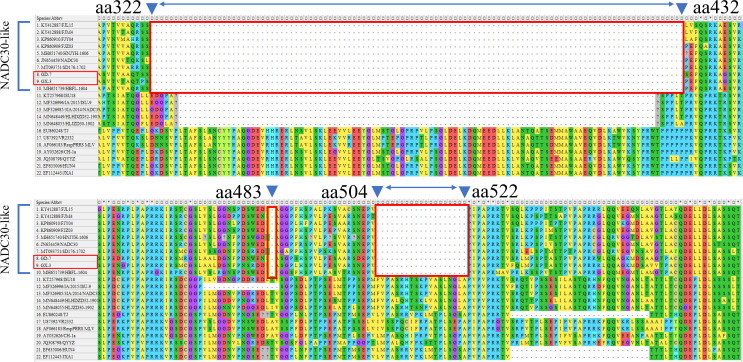
Amino acid deletion pattern in the NSP2 gene. The alignment of GD-7 and GX-3 is marked in red.

The results of genome sequencing of isolated strains were spliced using DNASTAR software to construct a phylogenetic evolutionary tree based on GP5 and the whole genome. The phylogenetic tree constructed based on the GP5 gene demonstrated that both GD-7 and GX-3 were clustered in the same branch with NADC30. Similarly, the evolutionary analysis tree constructed based on the whole genome revealed that GD-7 and GX-3 were similarly clustered. The strains were found to be clustered with NADC30 and to belong to the same lineage 1.8 (Fig. 3A and B). Furthermore, homology comparisons of the genomes revealed that the two strains were more homologous to NADC30-like PRRSV(Fig. 3C). A comparison of the NSP2 amino acid sequences of GD-7 and GX-3 with the reference strains revealed that the two isolates exhibited the same amino acid deletion pattern of “111 + 1 + 19” in the NSP2 region as that of NADC30 strains (Fig. 4). Consequently, it was determined that GD-7 and GX-3 isolates were NADC30-like. The GD-7 and GX-3 isolates were determined to be NADC30-like PRRSV.

**Fig 5:**
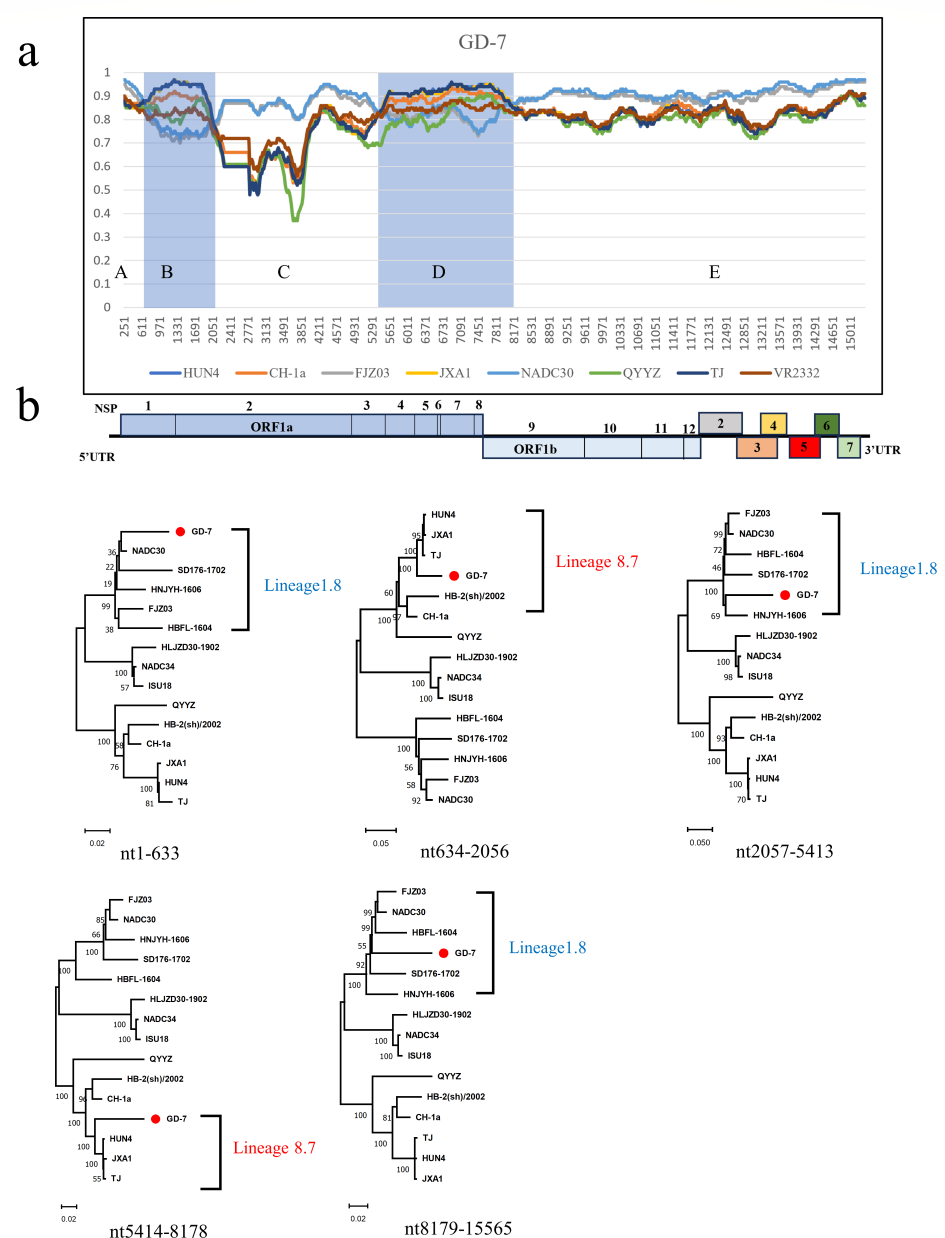
(a) Recombination analysis of the GD-7 strain using Simplot software, the blue-shaded region is the minor parental region and the rest is the major parental region. Below the similarity plot is a full-genome structure, with reference to CH-1a, in which the positions and boundaries of the major ORFs, nsp-encoding genes within ORF1a and ORF2b, and gaps are shown. (b) Phylogenetic trees were constructed based on GD-7 recombination intervals A, B, C, D, and E. Phylogenetic trees were constructed using the distance-based neighbor-joining method with 1,000 bootstrap replicates in MEGA11.

Recombination analysis of GD-7 and GX-3 using RDP4 with Simplot software revealed that both strains showed recombination signals. Four recombination breakpoints were identified in the complete sequence of GD-7 strain, located in NSP1(nt634), NSP2(nt2056), NSP3(nt5414), and NSP9(nt8178). These breakpoints formed five recombination intervals: intervals A (5′UTR-NSP1: nt1-633), B (NSP1-NSP2: nt634-2056), C (NSP2-NSP3: nt2057-5413), D (NSP3-NSP9: nt5413-8178), and E (NSP9-3′UTR: nt8179-15565), in which NADC30 was the major parental strain, and the representative strain of HP-PRRSV, strain TJ, was the minor parental strain (Fig. 5a; Table 2). Furthermore, phylogenetic analysis showed that recombination intervals B and D clustered together with the TJ strain and were grouped under Lineage 8 (Fig. 5b). While recombination intervals A, C, and E were grouped under Lineage 1.8 and clustered with NADC30 (Fig. 5b).

**TABLE 2:** Summary of crossover events in GD-7 and GX-3 stains identified by RDP4

Recombined virus	Parental virus		Score for the seven detection methods embedded in RDP4						
	Major	Minor	RDP	GENECONV	Boot scan	Max Chi	Chimaera	Si scan	3Seq
GD-7	NADC30	TJ	2.814 × 10^−84^	2.995 × 10^−71^	4.111 × 10^−84^	2.457 × 10^−31^	4.042 × 10^−33^	6.516 × 10^−37^	6.217 × 10^−14^
	HNJYH-1606	HUN4	4.787 × 10^−85^	-	2.422 × 10^−84^	5.496 × 10^−29^	1.651 × 10^−32^	1.440 × 10^−33^	1.865 × 10^−13^
GX-3	NADC30	TJ	1.122 × 10^−56^	9.430 × 10^−27^	4.221 × 10^−55^	3.799 × 10^−23^	1.522 × 10^−24^	1.432 × 10^−17^	3.774 × 10^−13^
	NADC30	JXA1	2.676 × 10^−75^	3.349 × 10^−62^	3.484 × 10^−73^	4.106 × 10^−23^	1.023 × 10^−25^	5.812 × 10^−28^	3.774 × 10^−13^

**Fig 6:**
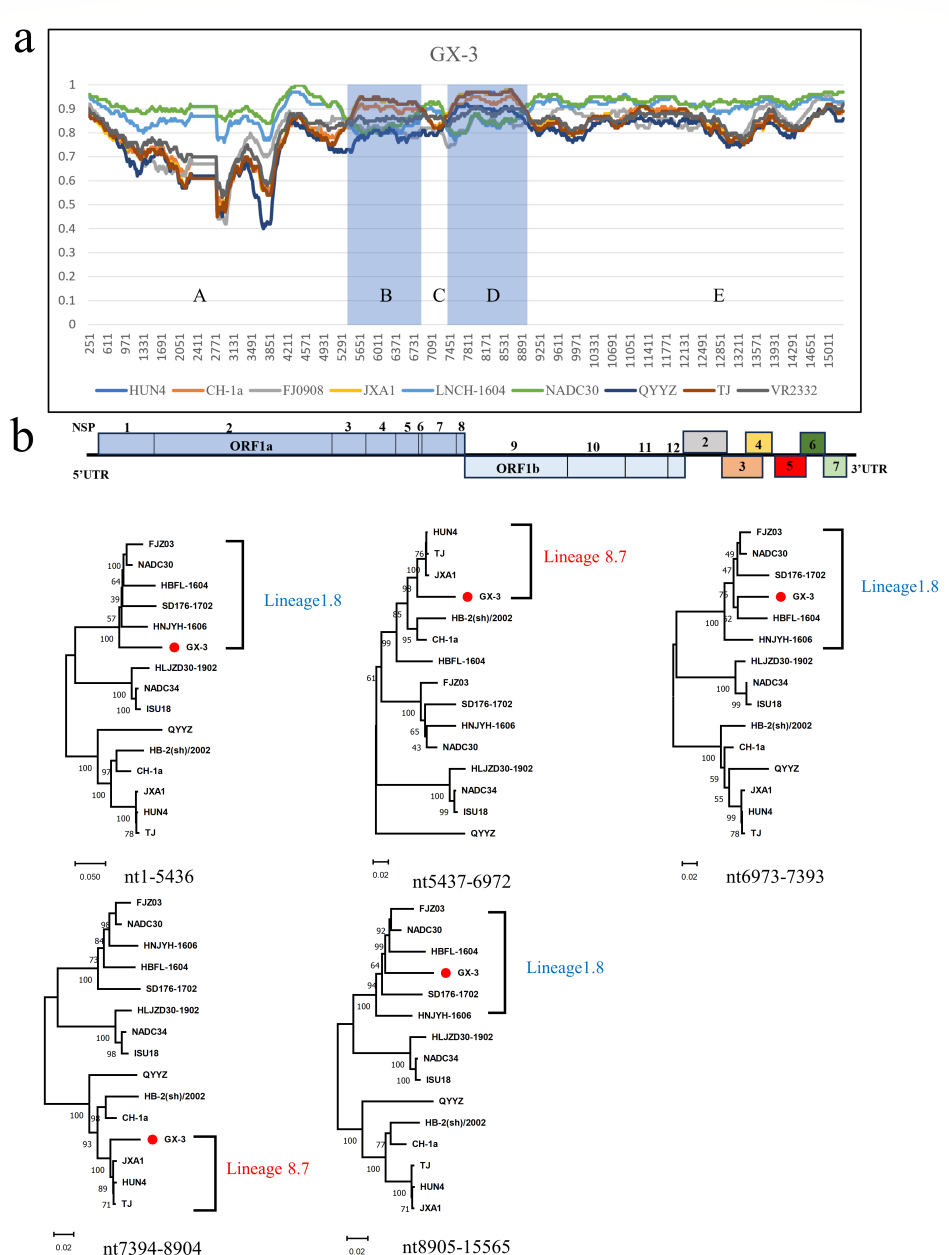
(a) Recombination analysis of the GX-3 strain using Simplot software, the blue-shaded region is the minor parental region and the rest is the major parental region. Below the similarity plot is a full-genome structure, with reference to CH-1a, in which the positions and boundaries of the major ORFs, nsp-encoding genes within ORF1a and ORF2b, and gaps are shown. (b) Phylogenetic trees were constructed based on GX-3 recombination intervals A, B, C, D, and E. Phylogenetic trees were constructed using the distance-based neighbor-joining method with 1,000 bootstrap replicates in MEGA11.

Four recombination breakpoints were also found in the complete sequence of the GX-3 strain, located in NSP3 (nt5437), NSP7 (nt6972 and nt7394), and NSP9 (nt8905). The four recombination breakpoints divided GX-3 into five recombination intervals: interval A (5′UTR-NSP3: nt1-5436), B (NSP3-NSP7: nt5437-6972), C (NSP7: nt6973-7393), D (NSP7-NSP9: nt7394-8904), and interval E (NSP9-3′UTR: 8905–15565), where NADC30 was the primary parent, and TJ and JXA1 strains were the secondary parents. (Fig. 6a; Table 2). Phylogenetic analysis revealed that interval B clustered with TJ and interval D clustered with JXA1, which belonged to Lineage 8 (Fig. 6b), and intervals A, C, and E clustered together with NADC30 and belonged to Lineage 1.8 (Fig. 6b). The above results indicated that both GD-7 and GX-3 were new strains formed by natural recombination of NADC30-like PRRSV and HP-PRRSV.

### Pathogenicity in piglets

**Fig 7:**
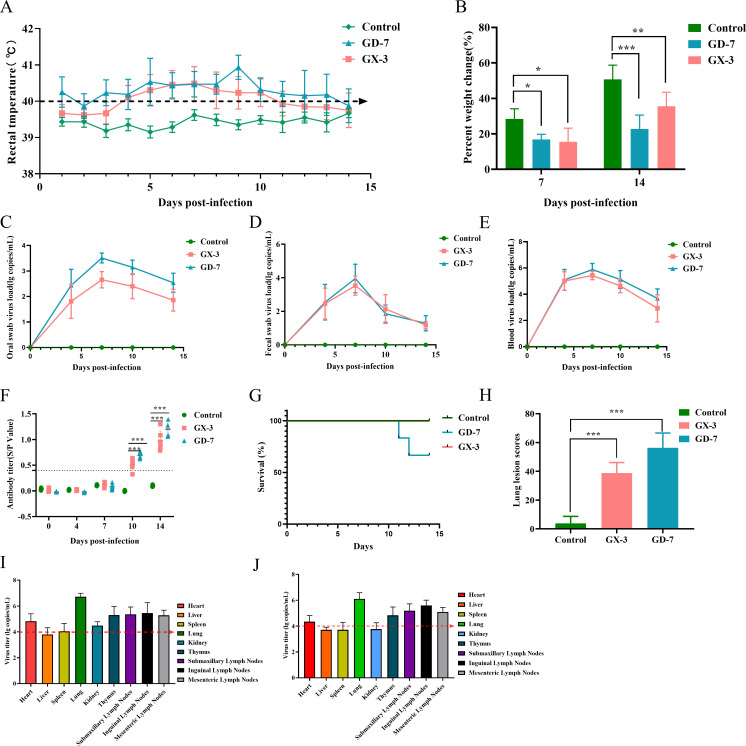
Pathogenicity results in piglets. (**A**) Body temperature change of pigs in each group after challenge. (**B**) Body weight gains of each group during the challenge study. (**C–E**): Viral load detection in blood, oral swabs, and pharyngeal swabs. (**F**) PRRSV-specific antibody level was detected in each group during the challenge study. (**G**) Ocular lesion scores of the lungs in each group. (**H**) Survival curves of piglets in each group. (**I**) The toxic load of various organs and tissues in the GD-7 group. (**H**) The toxic load of various organs and tissues in the GX-3 group. The significant difference is marked with the asterisk, ***P < 0.001, **P < 0.01, and *P < 0.05.

The GD-7 strain and GX-3 strain both manifested distinct clinical symptoms, including rapid breathing, lethargy, and anorexia. Moreover, the GD-7-inoculated group exhibited fever symptoms as early as 1 dpi, with the average body temperature of GD-7-inoculated piglets consistently surpassing 40°C throughout the experimental period, reaching its peak at 9 dpi. Conversely, piglets in the GX-3 inoculated group exhibited fever symptoms from 4 dpi to 10 dpi, followed by a declining trend in body temperature (Fig. 7A). The weight results indicated that the percentage increase in body weight for both the GD-7-inoculated group and GX-3-inoculated group piglets was lower than that of the control group (average increase: 50.8%) (Fig. 7B).

Oral swabs, fecal swabs, and anterior vena cava blood were collected at days 0, 3, 7, 10, and 14 for detoxification monitoring, viremia monitoring, and N protein antibody-level monitoring. Results showed that the virus was detectable in the throat and rectal swabs of all piglets in both the GD-7 and GX-3 groups at 4 dpi, indicating the initiation of viral shedding. Moreover, peak shedding occurred at 7 dpi, followed by a declining trend (Fig. 7C and D). Viral load monitoring in peripheral venous blood indicated that both the GD-7 and GX-3 groups reached peak viremia at 7 dpi, with the GD-7 group exhibiting a higher viremia level (10^5.9^ copies/mL) compared to the GX-3 group (10^5.4^ copies/mL) (Fig. 7D), suggesting a faster replication of the GD-7 strain in piglets. Serum PRRSV N antibody detection revealed that both strains induced PRRSV antibody seroconversion on the 10th day; however, GD-7-challenged piglets exhibited higher antibody levels than the GX-3-challenged group at 10 dpi. Importantly, throughout the entire experimental period, neither the blank control group nor the GX-3 challenged piglets experienced mortality, whereas two piglets from the GD-7-challenged group died at 11 dpi and 12 dpi.

**Fig 8:**
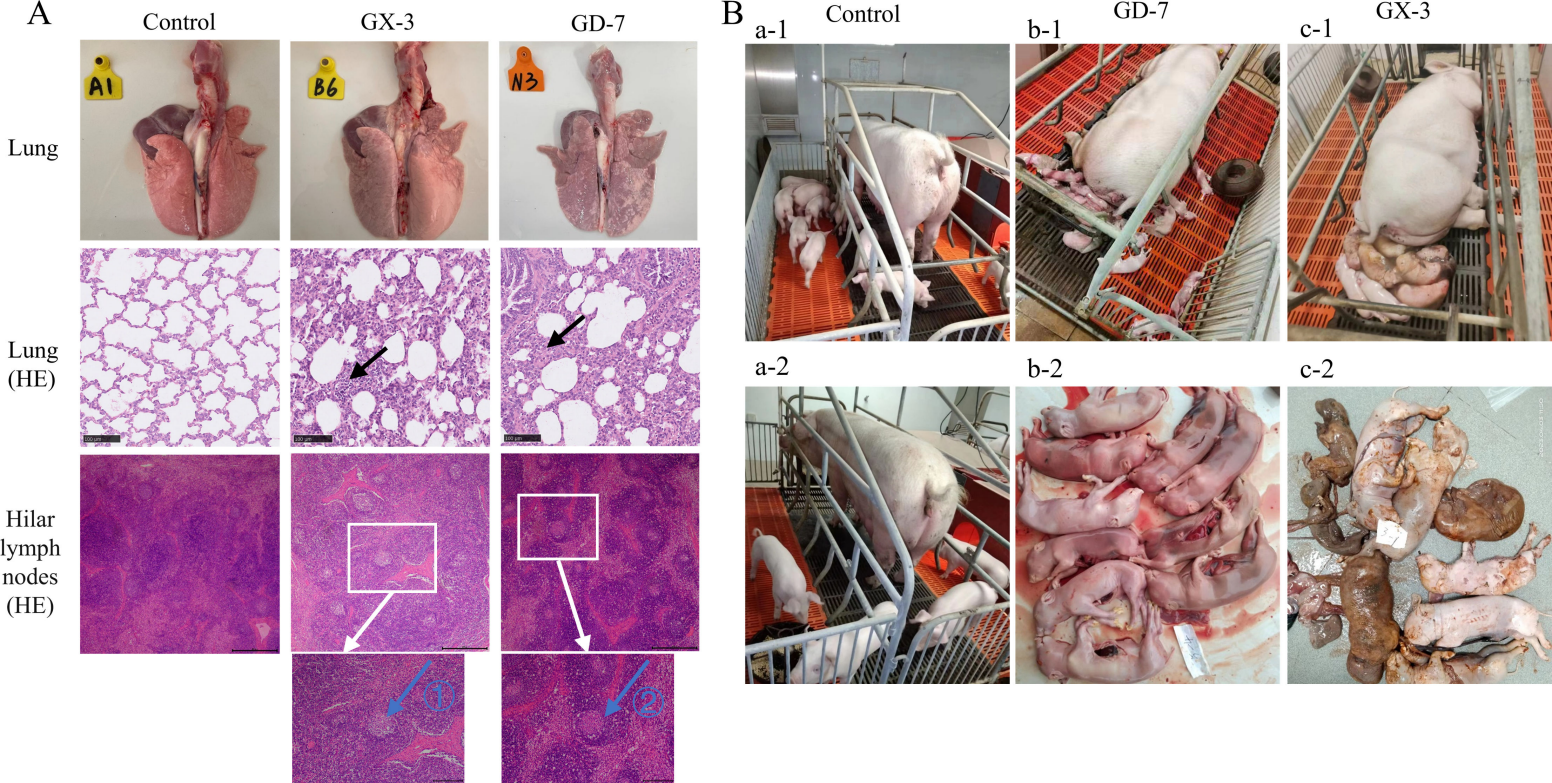
(A) Ocular lesions of the lungs and HE staining results of lung and Hilar lymphoid tissues; it shows interstitial thickening and inflammatory infiltration by black arrows; sparsely arranged lymphoid cells by blue arrow ①; and lymphocyte necrosis by blue arrow ②. (**B**) Clinical production results of sows in each group: a-1 and a-2 are control sows and their farrowing piglets; b-1, and b-2 are GD-7 sows and their aborting piglets; c-1 and c-2 are GX-3 sows and their farrowing mummified piglets.

All piglets were euthanized 14 days after inoculation and tested for organ viral load, revealing that the highest viral load was detected in the lungs (≥10^6^ copies/mL), followed by the lymphoid tissue and thymus (10^4^–10^6^ copies/mL). Furthermore, the GD-7-inoculated group exhibited higher organ viral loads than the GX-3-inoculated group (Fig. 7I and J). Lung ocular lesion results indicated severe lesions in GD-7-inoculated piglets (Fig. 7G), with large solid areas observed, especially in the cardiac and apical lobes. By contrast, GX-3-inoculated piglets showed smaller solid areas in the apical and cardiac lobes, with edema detected in the interlobular lobes. Conversely, the control group exhibited normal lungs. In addition, both GX-3- and GD-7-inoculated piglets showed signs of interstitial pneumonia, including interstitial thickening and inflammatory infiltration, accompanied by alveolar wall infiltration by numerous macrophages and a small number of lymphocytes and neutrophils (Fig. 8A, indicated by black arrows). By contrast, the control group exhibited normal alveoli. H&E staining of hilar lymph nodes revealed that they were normal in the control group, whereas lymph nodes in the GD-7-inoculated group exhibited exudation of protein-like material and lymphocyte necrosis (Fig. 8A, indicated by blue arrows ②). In the GX-3-inoculated group, edema was observed in lymphoid tissues, and lymph nodes displayed sparsely arranged lymphocytes (Fig. 8A, indicated by blue arrows ①).

### Pathogenicity in gestating sows

**Fig 9:**
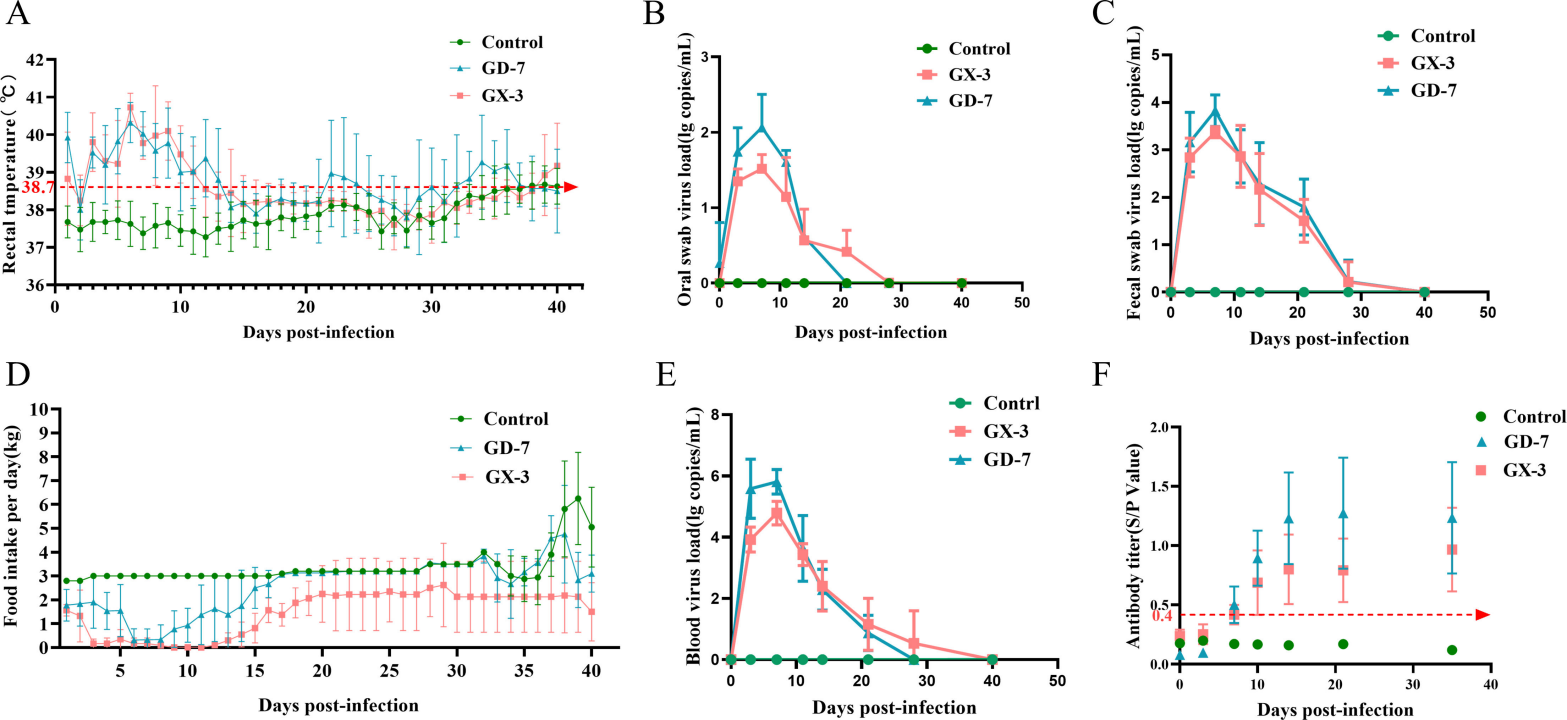
Pathogenicity results in sows. (**A**) Body temperature change of pigs in each group after the challenge. (**B and C**) Viral load detection oral swabs and fecal swabs. (**D**) Feed intake test for each group of sows. (**E**) Viral load detection in blood. (**F**) PRRSV-specific antibody level was detected in each group during the challenge study.

The pathogenicity of GX-3 and GD-7 strains in pregnant sows was investigated by tapping the sows. The results revealed increased body temperature of pregnant sows, which lasted approximately 2 weeks post-tapping before returning to normal (Fig. 9A). The GX-3-inoculated sows displayed a reduction in feed intake in the later part of the experiment (Fig. 9D), with one sow in the GX-3 group ceasing to eat from day 26 post-attack until the end of the experiment.

During the experiment, one GD-7-inoculated sow died on day 14 post-attack, two underwent an abortion on day 28 post-attack, and only one gave birth normally, delivering four weak piglets. In the GX-3-inoculated group, two sows underwent an abortion on days 32 and 33 post-attack, respectively, with the remaining two sows giving birth to four and five piglets, respectively (Fig. 8B; Table 3). Monitoring of detoxification revealed higher detoxification through the anus than through the oropharynx, with peak detoxification observed on day 7 post-detoxification. GX-3-inoculated sows exhibited prolonged detoxification compared with GD-7-inoculated sows (Fig. 9B and C). Moreover, GX-3 strain inoculation led to prolonged viremia in sows (Fig. 9E). Serum analysis of N protein antibodies revealed seroconversion in all sows on day 10 post-attack (Fig. 9F).

**TABLE 3:** Farrowing data of sows during the experiment

Groups	Control	GD-7	GX-3
Challenge virus	DMEM	Virus cultures	Virus cultures
Piglets, number per litter			
Live-born	12/4/7/9	0/4/0/0	0/0/5/4
Healthy piglets	12/4/7/9	0/0/0/0	0/0/5/3
Weak offspring	0/0/0/0	0/4/0/0	0/0/0/1
Stillborn	0/1/0/1	14/4/0/13	11/1/2/3
Mummified	0/0/0/0	0/3/0/0	0/13/1/8
Total born	12/5/7/10	14/11/0/13	11/14/8/15

## DISCUSSION

Since its discovery in China in 2013 (8, 11, 12), NADC30-like PRRSV has become the dominant PRRSV strain, posing challenges to PRRSV prevention and control. The robust recombination and mutation abilities of the strain coupled with the coexistence of multiple PRRSVs in farms contribute to its prevalence (13). Existing commercialized vaccines lack efficacy in cross-protection against NADC30-like PRRSVs, further exacerbating the challenges associated with the control of PRRSV.

In April 2023, we analyzed lung tissue specimens of piglets suspected to be infected with PRRSV from a pig farm in Guangdong and another in Guangxi. We found that sows of the two farms had serious abortions, and the mortality rate of piglets was significantly high. Moreover, isolation and identification of the virulent strains, whole-genome analysis, and pathogenicity analysis revealed that both strains clustered with NADC30 in Lineage 1.8 and had a 119 + 1 + 19 amino acid deletion pattern in the NSP2 region, which indicated that GD-7 and GX-3 belonged to the NADC30-like PRRSV. Recombination is the primary driver of NADC30-like PRRSV pathogenicity and genetic diversity in China (14, 15), but mechanisms underlying PRRSV recombination remain unclear. However, recombination analysis of isolated NADC30-like PRRSV strains revealed recombination breakpoints in 5′ untranslated regions (UTRs), including NSP1–9, NSP12, ORF3, and ORF5–3′ UTRs (15–17). Interestingly, our recombination analysis of GD-7 and GX-3 revealed that both strains recombined with HP-PRRSV in the NSP5-NSP9 interval. We will continue our work on the epidemiology of NADC30-like PRRSV and pay attention to the frequency of this recombination.

Natural recombination events between NADC30-like PRRSV and HP-PRRSV, such as PRRSV2/CN/FJGD01/2021, SC-d, FJSX15, and BL2019, have recently been reported. Differences in the recombination region between these strains and HP-PRRSV result in varied pathogenicity, which is often intermediate between the parental strains (7, 14, 18, 19). We developed a systematic model of pathogenicity in piglets and sows to further investigate the pathogenicity of NADC30-like PRRSV and HP-PRRSV. Pathogenicity tests revealed that both strains caused clinical signs in piglets, including mild coughing, anorexia, and reduced weight gain, control piglets showed no such clinical signs. The challenged group showed typical PRRSV lesions, such as solid lung lesions and petechiae. Histopathology indicated decreased lymphocyte count with interstitial pneumonia. Survival curves of each group showed that two piglets died in the GD-7-inoculated group, whereas no mortality was reported in the control and GX-3-inoculated groups. It is worth noting that GX-3 caused fever and respiratory disease in piglets without inducing mortality, consistent with the typical pathogenicity of NADC30-like PRRSVs (20–22). Conversely, the GD-7 strain exhibited higher pathogenicity, exceeding that of most NADC30-like PRRSV strains (7, 14, 18), which was similar to the results of piglets infected with other HP-PRRSV strains (23, 24), as evidenced by the death of two GD-7-inoculated piglets. Moreover, both isolates had a significant impact on the reproductive abilities of sows, resulting in stillbirths, mummified fetuses, abortions, and even the death of one pregnant sow in the GD-7 group. Notably, the GX-3 strain not only caused abortions in sows but also resulted in the delivery of more mummified fetuses.

In this study, we found that both GD-7 and GX-3 strains showed relatively stronger pathogenicity compared to normal NADC30 strains, and the pathogenicity of GD-7 strain was close to that of HP-PRRSV, which we speculated was due to the recombinant fragments being derived from HP-PRRSV. It was found by recombinant analysis of the strains that both GD-7 and GX-3 strains were recombined from NADC30 and HP-PRRSV strains, and the recombined regions were non-structural protein regions, and no recombination was found in the structural protein regions. Numerous studies have shown that nonstructural proteins play an important role in viral pathogenic and in the antagonism of PRRSV of natural host immunity (25). PRRSV NSP1 is the most potent IFN antagonist (26). In the nonstructural protein precursor polyprotein pp1a and pplab addition labor, NSP4 acts as an important replicative and transcriptional enzyme that cuts the polyproteins during PRRSV proliferation. NSP4 is also responsible for NSP3-NSP12 cleavage. A previous study that cloned highly pathogenic and low pathogenic PRRSV isolates using reverse genetics and exchange of gene fragments between the two strains revealed that NSP9 and NSP10 were the key virulence factors of HP-PRRSV *in vivo* (27). PRRSV ORF1b encodes NSP9, which is an RNA-dependent RNA polymerase (RdRp). RdRp is essential for genome replication and subgenome synthesis and plays a crucial role in viral replication (28). We hypothesize that the recombination of genomic fragments is responsible for the greater pathogenicity of GD-7 versus GX-3 strains in piglets and sows.

### Conclusion

In the present study, we identified and isolated GD-7 and GX-3, which are both natural recombinant strains of NADC30-like and HP-PRRSV genomes. Although the origins of the two strains differ, they both strains recombined with HP-PRRSV in the NSP3-NSP9 regions. The results of the piglet pathogenicity model showed that GD-7 caused the death of piglets and was more pathogenic than GX-3. The results of the pathogenicity model for pregnant sows showed that GD-7 strains not only caused severe abortion but also induced a 25% lethality rate in sows. GX-3 strains caused abortions and the delivery of mummified fetuses, which seriously affected the reproductive performance of sows. These findings highlight the urgency of evolving and responding to changing PRRSV strains to develop effective immunization and control strategies. Furthermore, this study provides a baseline reference for the epidemiology of PRRSV and guidance for prevention and control.

## Data Availability

The PRRSV genome sequences obtained in this study were deposited in the GenBank database under the accession numbers OR711915(GD-7) and OR582383(GX-3).
